# (2,2′-Bipyridine-κ^2^ *N*,*N*′)(4-hydr­oxy-2-oxidobenzaldehyde thio­semicar­ba­zon­ato-κ^3^ *O* ^2^,*N* ^1^,*S*)zinc(II)

**DOI:** 10.1107/S1600536808043973

**Published:** 2009-01-08

**Authors:** Kong Wai Tan, Chew Hee Ng, Mohd Jamil Maah, Seik Weng Ng

**Affiliations:** a Department of Chemistry, University of Malaya, 50603 Kuala Lumpur, Malaysia; b Faculty of Engineering and Science, Universiti Tunku Abdul Rahman, 53300 Kuala Lumpur, Malaysia

## Abstract

The Zn^II^ atom in the title compound, [Zn(C_8_H_7_N_3_O_2_S)(C_10_H_8_N_2_)], is *N*,*N*′-chelated by the heterocycle and *N*,*O*,*S*-chelated by the doubly deprotonated Schiff base ligand in a distorted square-pyramidal environment. O—H⋯O and N—H⋯N hydrogen bonds link adjacent mol­ecules into a layer structure.

## Related literature

For the square-pyramidal 1,10-phenanthroline adduct, which exists as a monohydrated DMSO disolvate, see: Tan *et al.* (2009 ▶).
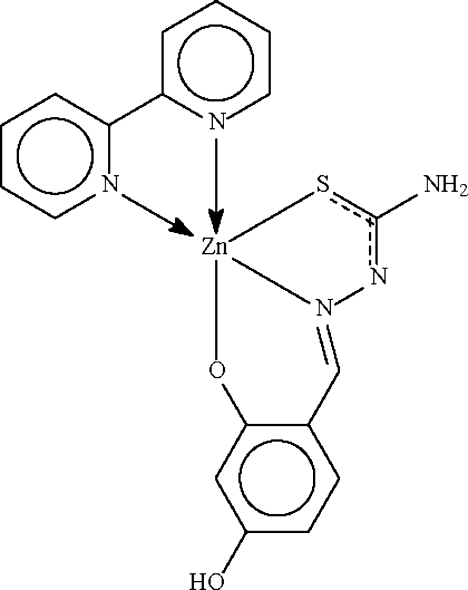


## Experimental

### 

#### Crystal data

[Zn(C_8_H_7_N_3_O_2_S)(C_10_H_8_N_2_)]
*M* *_r_* = 430.78
Monoclinic, 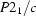

*a* = 16.1256 (4) Å
*b* = 7.0478 (2) Å
*c* = 17.6387 (5) Å
β = 113.646 (2)°
*V* = 1836.33 (9) Å^3^
*Z* = 4
Mo *K*α radiation
μ = 1.48 mm^−1^
*T* = 100 (2) K
0.10 × 0.04 × 0.02 mm

#### Data collection

Bruker SMART APEX diffractometer
Absorption correction: multi-scan (*SADABS*; Sheldrick, 1996 ▶) *T*_min_ = 0.867, *T*_max_ = 0.971
15773 measured reflections
4191 independent reflections
2919 reflections with *I* > 2σ(*I*)
*R*_int_ = 0.086

#### Refinement

*R*[*F* ^2^ > 2σ(*F* ^2^)] = 0.062
*wR*(*F* ^2^) = 0.195
*S* = 1.04
4191 reflections
245 parameters
24 restraints
H-atom parameters constrained
Δρ_max_ = 0.88 e Å^−3^
Δρ_min_ = −0.96 e Å^−3^

### 

Data collection: *APEX2* (Bruker, 2007 ▶); cell refinement: *SAINT* (Bruker, 2007 ▶); data reduction: *SAINT*; program(s) used to solve structure: *SHELXS97* (Sheldrick, 2008 ▶); program(s) used to refine structure: *SHELXL97* (Sheldrick, 2008 ▶); molecular graphics: *X-SEED* (Barbour, 2001 ▶); software used to prepare material for publication: *publCIF* (Westrip, 2009 ▶).

## Supplementary Material

Crystal structure: contains datablocks I, global. DOI: 10.1107/S1600536808043973/tk2348sup1.cif

Structure factors: contains datablocks I. DOI: 10.1107/S1600536808043973/tk2348Isup2.hkl

Additional supplementary materials:  crystallographic information; 3D view; checkCIF report

## Figures and Tables

**Table 1:** Hydrogen-bond geometry (Å, °)

*D*—H⋯*A*	*D*—H	H⋯*A*	*D*⋯*A*	*D*—H⋯*A*
O2—H2⋯O1^i^	0.84	1.85	2.625 (5)	153
N3—H32⋯N2^ii^	0.88	2.15	2.936 (7)	148

Symmetry codes: (i) 
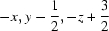
; (ii) 
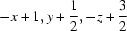
.

**Fig. 1.**
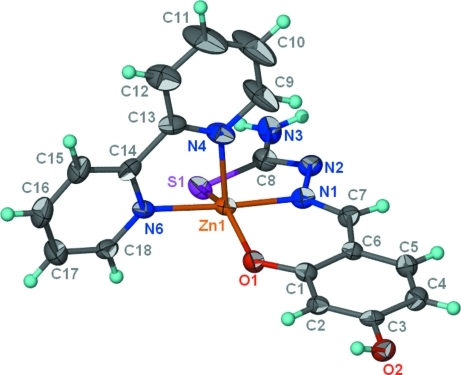
Thermal ellipsoid (Barbour, 2001) plot of Zn(C8H7N3O2S)(C10H8N2) at the 70% probability level. Hydrogen atoms are drawn as spheres of arbitrary radii.

## supplementary crystallographic information

### Experimental

Zinc acetate monohydrate (0.22 g, 1 mmol), 2,4-dihydroxybenzaldehyde thiosemicarbazone (0.21 g, 1 mmol) and 2,2'-bipyridine (0.16 g, 1 mmol) were heated in ethanol (40 ml). The compound that precipitated upon heating for 30 min was collected and recrystallized from DMF.

### Refinement

Hydrogen atoms were placed in calculated positions (C–H 0.95, N–H 0.88, O–H 0.84 Å) and were included in the refinement in the riding model approximation, with *U*(H) set to 1.2–1.5*U*(*C*,*N*,*O*).

The four carbon atoms of one of the two rings of the 2,2'-bipyridine molecule showed somewhat large anisotropic temperature factors. These were restrained to be nearly isotropic.

### Crystal data

**Table:**

[Zn(C_8_H_7_N_3_O_2_S)(C_10_H_8_N_2_)]	*F*(000) = 880
*M**_r_* = 430.78	*D*_x_ = 1.558 Mg m^−^^3^
Monoclinic, *P*2_1_/*c*	Mo *K*α radiation, λ = 0.71073 Å
Hall symbol: -P 2ybc	Cell parameters from 3053 reflections
*a* = 16.1256 (4) Å	θ = 2.3–26.2°
*b* = 7.0478 (2) Å	µ = 1.48 mm^−^^1^
*c* = 17.6387 (5) Å	*T* = 100 K
β = 113.646 (2)°	Prism, yellow
*V* = 1836.33 (9) Å^3^	0.10 × 0.04 × 0.02 mm
*Z* = 4	

### Data collection

**Table:**

Bruker SMART APEX diffractometer	4191 independent reflections
Radiation source: fine-focus sealed tube	2919 reflections with *I* > 2σ(*I*)
graphite	*R*_int_ = 0.086
ω scans	θ_max_ = 27.5°, θ_min_ = 1.4°
Absorption correction: multi-scan (*SADABS*; Sheldrick, 1996)	*h* = −20→20
*T*_min_ = 0.867, *T*_max_ = 0.971	*k* = −9→9
15773 measured reflections	*l* = −21→22

### Refinement

**Table:**

Refinement on *F*^2^	Primary atom site location: structure-invariant direct methods
Least-squares matrix: full	Secondary atom site location: difference Fourier map
*R*[*F*^2^ > 2σ(*F*^2^)] = 0.062	Hydrogen site location: inferred from neighbouring sites
*wR*(*F*^2^) = 0.195	H-atom parameters constrained
*S* = 1.04	*w* = 1/[σ^2^(*F*_o_^2^) + (0.1071*P*)^2^ + 3.3408*P*] where *P* = (*F*_o_^2^ + 2*F*_c_^2^)/3
4191 reflections	(Δ/σ)_max_ = 0.001
245 parameters	Δρ_max_ = 0.88 e Å^−^^3^
24 restraints	Δρ_min_ = −0.96 e Å^−^^3^

### Fractional atomic coordinates and isotropic or equivalent isotropic displacement parameters (Å^2^)

**Table:**

	*x*	*y*	*z*	*U*_iso_*/*U*_eq_	
Zn1	0.26010 (4)	0.61341 (9)	0.78222 (4)	0.0186 (2)	
S1	0.35671 (10)	0.8297 (2)	0.75159 (9)	0.0239 (3)	
O1	0.1393 (2)	0.4912 (5)	0.7423 (2)	0.0221 (8)	
O2	−0.0804 (3)	−0.0017 (6)	0.6388 (2)	0.0238 (8)	
H2	−0.0941	0.0325	0.6780	0.036*	
N1	0.2953 (3)	0.4436 (7)	0.7058 (3)	0.0213 (10)	
N2	0.3774 (3)	0.4754 (7)	0.6981 (3)	0.0226 (10)	
N3	0.4867 (3)	0.6892 (7)	0.7112 (3)	0.0276 (11)	
H31	0.5135	0.6021	0.6932	0.033*	
H32	0.5107	0.8029	0.7242	0.033*	
N4	0.3299 (3)	0.4781 (7)	0.8970 (3)	0.0276 (11)	
N6	0.2340 (3)	0.7942 (6)	0.8656 (3)	0.0186 (9)	
C1	0.1130 (4)	0.3300 (8)	0.7005 (3)	0.0198 (11)	
C2	0.0305 (3)	0.2459 (8)	0.6918 (3)	0.0192 (11)	
H2A	−0.0049	0.3053	0.7170	0.023*	
C3	−0.0009 (4)	0.0790 (8)	0.6476 (3)	0.0199 (11)	
C4	0.0493 (4)	−0.0115 (8)	0.6105 (3)	0.0250 (12)	
H4	0.0276	−0.1248	0.5798	0.030*	
C5	0.1305 (4)	0.0642 (8)	0.6187 (3)	0.0246 (12)	
H5	0.1644	0.0017	0.5927	0.030*	
C6	0.1660 (3)	0.2315 (7)	0.6641 (3)	0.0182 (10)	
C7	0.2514 (4)	0.2930 (8)	0.6672 (3)	0.0210 (11)	
H7	0.2788	0.2180	0.6388	0.025*	
C8	0.4083 (4)	0.6481 (8)	0.7189 (3)	0.0233 (12)	
C9	0.3807 (6)	0.3222 (11)	0.9082 (5)	0.0486 (19)	
H9	0.3852	0.2632	0.8615	0.058*	
C10	0.4268 (7)	0.2446 (14)	0.9857 (5)	0.068 (3)	
H10	0.4631	0.1346	0.9924	0.082*	
C11	0.4195 (6)	0.3282 (13)	1.0529 (5)	0.059 (2)	
H11	0.4503	0.2752	1.1065	0.071*	
C12	0.3680 (5)	0.4878 (11)	1.0429 (4)	0.0390 (16)	
H12	0.3628	0.5480	1.0890	0.047*	
C13	0.3235 (4)	0.5594 (9)	0.9635 (3)	0.0246 (12)	
C14	0.2655 (4)	0.7348 (8)	0.9444 (3)	0.0235 (12)	
C15	0.2450 (5)	0.8306 (10)	1.0037 (4)	0.0345 (15)	
H15	0.2672	0.7861	1.0592	0.041*	
C16	0.1917 (5)	0.9915 (11)	0.9804 (4)	0.0429 (18)	
H16	0.1754	1.0573	1.0194	0.051*	
C17	0.1624 (5)	1.0556 (9)	0.9000 (4)	0.0356 (15)	
H17	0.1276	1.1684	0.8831	0.043*	
C18	0.1848 (4)	0.9525 (8)	0.8448 (4)	0.0242 (12)	
H18	0.1643	0.9961	0.7893	0.029*	

### Atomic displacement parameters (Å^2^)

**Table:**

	*U*^11^	*U*^22^	*U*^33^	*U*^12^	*U*^13^	*U*^23^
Zn1	0.0165 (3)	0.0207 (3)	0.0182 (3)	0.0020 (2)	0.0065 (2)	0.0029 (2)
S1	0.0208 (7)	0.0196 (7)	0.0340 (8)	0.0012 (5)	0.0139 (6)	0.0039 (5)
O1	0.0178 (19)	0.021 (2)	0.028 (2)	−0.0017 (15)	0.0101 (16)	−0.0024 (16)
O2	0.0186 (19)	0.027 (2)	0.025 (2)	−0.0039 (16)	0.0079 (16)	−0.0022 (16)
N1	0.018 (2)	0.026 (2)	0.021 (2)	−0.0014 (19)	0.0095 (19)	0.0022 (18)
N2	0.014 (2)	0.030 (3)	0.024 (2)	−0.0033 (19)	0.0094 (19)	−0.002 (2)
N3	0.022 (2)	0.025 (2)	0.043 (3)	−0.006 (2)	0.021 (2)	−0.005 (2)
N4	0.027 (3)	0.033 (3)	0.024 (2)	0.009 (2)	0.012 (2)	0.012 (2)
N6	0.016 (2)	0.019 (2)	0.019 (2)	0.0003 (17)	0.0052 (18)	0.0038 (17)
C1	0.020 (3)	0.019 (3)	0.019 (3)	0.002 (2)	0.007 (2)	0.004 (2)
C2	0.016 (2)	0.024 (3)	0.018 (2)	0.002 (2)	0.007 (2)	0.002 (2)
C3	0.015 (2)	0.023 (3)	0.018 (2)	0.001 (2)	0.002 (2)	0.006 (2)
C4	0.027 (3)	0.026 (3)	0.022 (3)	−0.002 (2)	0.010 (2)	0.000 (2)
C5	0.024 (3)	0.028 (3)	0.023 (3)	0.003 (2)	0.010 (2)	−0.003 (2)
C6	0.018 (3)	0.018 (3)	0.018 (2)	0.002 (2)	0.006 (2)	0.0031 (19)
C7	0.022 (3)	0.025 (3)	0.018 (3)	0.000 (2)	0.009 (2)	−0.001 (2)
C8	0.022 (3)	0.026 (3)	0.025 (3)	0.001 (2)	0.013 (2)	0.003 (2)
C9	0.059 (4)	0.049 (4)	0.048 (4)	0.032 (4)	0.032 (4)	0.022 (3)
C10	0.085 (6)	0.075 (5)	0.054 (5)	0.054 (5)	0.037 (4)	0.031 (4)
C11	0.065 (5)	0.076 (5)	0.039 (4)	0.037 (4)	0.024 (4)	0.032 (4)
C12	0.039 (4)	0.051 (4)	0.032 (3)	0.010 (3)	0.019 (3)	0.015 (3)
C13	0.020 (3)	0.031 (3)	0.021 (3)	0.000 (2)	0.006 (2)	0.007 (2)
C14	0.022 (3)	0.027 (3)	0.019 (3)	−0.007 (2)	0.005 (2)	−0.003 (2)
C15	0.042 (4)	0.036 (4)	0.021 (3)	0.001 (3)	0.008 (3)	−0.007 (2)
C16	0.053 (5)	0.041 (4)	0.029 (3)	0.004 (3)	0.011 (3)	−0.014 (3)
C17	0.040 (4)	0.028 (3)	0.034 (3)	0.010 (3)	0.010 (3)	−0.002 (3)
C18	0.020 (3)	0.023 (3)	0.023 (3)	−0.001 (2)	0.002 (2)	−0.001 (2)

### Geometric parameters (Å, °)

**Table:**

Zn1—O1	1.983 (4)	C4—C5	1.367 (8)
Zn1—N1	2.045 (5)	C4—H4	0.9500
Zn1—N4	2.109 (5)	C5—C6	1.412 (8)
Zn1—N6	2.112 (5)	C5—H5	0.9500
Zn1—S1	2.3911 (15)	C6—C7	1.423 (7)
S1—C8	1.746 (6)	C7—H7	0.9500
O1—C1	1.329 (7)	C9—C10	1.381 (10)
O2—C3	1.354 (6)	C9—H9	0.9500
O2—H2	0.8400	C10—C11	1.370 (12)
N1—C7	1.306 (7)	C10—H10	0.9500
N1—N2	1.403 (6)	C11—C12	1.367 (10)
N2—C8	1.310 (7)	C11—H11	0.9500
N3—C8	1.355 (7)	C12—C13	1.387 (8)
N3—H31	0.8800	C12—H12	0.9500
N3—H32	0.8800	C13—C14	1.505 (8)
N4—C9	1.338 (8)	C14—C15	1.393 (8)
N4—C13	1.346 (8)	C15—C16	1.382 (10)
N6—C18	1.333 (7)	C15—H15	0.9500
N6—C14	1.340 (7)	C16—C17	1.380 (9)
C1—C2	1.408 (7)	C16—H16	0.9500
C1—C6	1.437 (7)	C17—C18	1.373 (9)
C2—C3	1.389 (8)	C17—H17	0.9500
C2—H2A	0.9500	C18—H18	0.9500
C3—C4	1.385 (8)		
			
O1—Zn1—N1	90.29 (16)	C6—C5—H5	118.7
O1—Zn1—N4	102.37 (18)	C5—C6—C7	116.2 (5)
N1—Zn1—N4	100.70 (19)	C5—C6—C1	118.6 (5)
O1—Zn1—N6	93.82 (16)	C7—C6—C1	125.1 (5)
N1—Zn1—N6	175.77 (18)	N1—C7—C6	125.5 (5)
N4—Zn1—N6	77.46 (19)	N1—C7—H7	117.2
O1—Zn1—S1	146.42 (12)	C6—C7—H7	117.2
N1—Zn1—S1	81.14 (13)	N2—C8—N3	115.8 (5)
N4—Zn1—S1	111.10 (15)	N2—C8—S1	126.5 (4)
N6—Zn1—S1	95.92 (13)	N3—C8—S1	117.6 (4)
C8—S1—Zn1	92.73 (19)	N4—C9—C10	121.7 (7)
C1—O1—Zn1	128.1 (3)	N4—C9—H9	119.1
C3—O2—H2	109.5	C10—C9—H9	119.1
C7—N1—N2	114.5 (5)	C11—C10—C9	119.2 (7)
C7—N1—Zn1	125.7 (4)	C11—C10—H10	120.4
N2—N1—Zn1	119.5 (3)	C9—C10—H10	120.4
C8—N2—N1	112.9 (5)	C10—C11—C12	120.0 (7)
C8—N3—H31	120.0	C10—C11—H11	120.0
C8—N3—H32	120.0	C12—C11—H11	120.0
H31—N3—H32	120.0	C11—C12—C13	118.1 (7)
C9—N4—C13	118.5 (5)	C11—C12—H12	121.0
C9—N4—Zn1	124.9 (5)	C13—C12—H12	121.0
C13—N4—Zn1	116.5 (4)	N4—C13—C12	122.4 (6)
C18—N6—C14	118.9 (5)	N4—C13—C14	114.3 (5)
C18—N6—Zn1	125.2 (4)	C12—C13—C14	123.2 (6)
C14—N6—Zn1	115.8 (4)	N6—C14—C15	121.5 (6)
O1—C1—C2	119.8 (5)	N6—C14—C13	115.6 (5)
O1—C1—C6	123.2 (5)	C15—C14—C13	122.9 (5)
C2—C1—C6	117.0 (5)	C16—C15—C14	118.7 (6)
C3—C2—C1	122.2 (5)	C16—C15—H15	120.6
C3—C2—H2A	118.9	C14—C15—H15	120.6
C1—C2—H2A	118.9	C17—C16—C15	119.3 (6)
O2—C3—C4	117.3 (5)	C17—C16—H16	120.3
O2—C3—C2	122.4 (5)	C15—C16—H16	120.3
C4—C3—C2	120.3 (5)	C18—C17—C16	118.5 (6)
C5—C4—C3	119.2 (5)	C18—C17—H17	120.8
C5—C4—H4	120.4	C16—C17—H17	120.8
C3—C4—H4	120.4	N6—C18—C17	123.0 (5)
C4—C5—C6	122.6 (5)	N6—C18—H18	118.5
C4—C5—H5	118.7	C17—C18—H18	118.5
			
O1—Zn1—S1—C8	−95.8 (3)	C4—C5—C6—C1	−2.5 (8)
N1—Zn1—S1—C8	−18.8 (2)	O1—C1—C6—C5	−178.1 (5)
N4—Zn1—S1—C8	79.3 (2)	C2—C1—C6—C5	3.3 (7)
N6—Zn1—S1—C8	158.1 (2)	O1—C1—C6—C7	−0.7 (8)
N1—Zn1—O1—C1	15.3 (4)	C2—C1—C6—C7	−179.2 (5)
N4—Zn1—O1—C1	−85.8 (4)	N2—N1—C7—C6	−178.8 (5)
N6—Zn1—O1—C1	−163.8 (4)	Zn1—N1—C7—C6	7.3 (8)
S1—Zn1—O1—C1	89.5 (5)	C5—C6—C7—N1	180.0 (5)
O1—Zn1—N1—C7	−13.1 (5)	C1—C6—C7—N1	2.5 (9)
N4—Zn1—N1—C7	89.5 (5)	N1—N2—C8—N3	−178.7 (5)
S1—Zn1—N1—C7	−160.5 (5)	N1—N2—C8—S1	−1.0 (7)
O1—Zn1—N1—N2	173.2 (4)	Zn1—S1—C8—N2	17.3 (5)
N4—Zn1—N1—N2	−84.1 (4)	Zn1—S1—C8—N3	−165.0 (4)
S1—Zn1—N1—N2	25.8 (4)	C13—N4—C9—C10	−0.5 (12)
C7—N1—N2—C8	163.9 (5)	Zn1—N4—C9—C10	178.0 (7)
Zn1—N1—N2—C8	−21.8 (6)	N4—C9—C10—C11	0.7 (15)
O1—Zn1—N4—C9	92.1 (6)	C9—C10—C11—C12	−0.8 (16)
N1—Zn1—N4—C9	−0.5 (6)	C10—C11—C12—C13	0.6 (13)
N6—Zn1—N4—C9	−176.7 (6)	C9—N4—C13—C12	0.3 (10)
S1—Zn1—N4—C9	−85.1 (6)	Zn1—N4—C13—C12	−178.3 (5)
O1—Zn1—N4—C13	−89.4 (4)	C9—N4—C13—C14	179.5 (6)
N1—Zn1—N4—C13	177.9 (4)	Zn1—N4—C13—C14	0.9 (6)
N6—Zn1—N4—C13	1.8 (4)	C11—C12—C13—N4	−0.4 (10)
S1—Zn1—N4—C13	93.4 (4)	C11—C12—C13—C14	−179.5 (7)
O1—Zn1—N6—C18	−78.4 (4)	C18—N6—C14—C15	2.6 (8)
N4—Zn1—N6—C18	179.8 (5)	Zn1—N6—C14—C15	−173.4 (5)
S1—Zn1—N6—C18	69.4 (4)	C18—N6—C14—C13	−177.6 (5)
O1—Zn1—N6—C14	97.3 (4)	Zn1—N6—C14—C13	6.4 (6)
N4—Zn1—N6—C14	−4.6 (4)	N4—C13—C14—N6	−4.9 (7)
S1—Zn1—N6—C14	−114.9 (4)	C12—C13—C14—N6	174.3 (6)
Zn1—O1—C1—C2	167.2 (4)	N4—C13—C14—C15	174.9 (6)
Zn1—O1—C1—C6	−11.3 (7)	C12—C13—C14—C15	−5.9 (9)
O1—C1—C2—C3	179.0 (5)	N6—C14—C15—C16	−0.7 (10)
C6—C1—C2—C3	−2.4 (8)	C13—C14—C15—C16	179.5 (6)
C1—C2—C3—O2	−179.6 (5)	C14—C15—C16—C17	−1.7 (11)
C1—C2—C3—C4	0.3 (8)	C15—C16—C17—C18	2.3 (11)
O2—C3—C4—C5	−179.3 (5)	C14—N6—C18—C17	−2.0 (9)
C2—C3—C4—C5	0.7 (8)	Zn1—N6—C18—C17	173.5 (5)
C3—C4—C5—C6	0.4 (9)	C16—C17—C18—N6	−0.5 (10)
C4—C5—C6—C7	179.9 (5)		

### Hydrogen-bond geometry (Å, °)

**Table:**

*D*—H···*A*	*D*—H	H···*A*	*D*···*A*	*D*—H···*A*
O2—H2···O1^i^	0.84	1.85	2.625 (5)	153
N3—H32···N2^ii^	0.88	2.15	2.936 (7)	148

Symmetry codes: (i) −*x*, *y*−1/2, −*z*+3/2; (ii) −*x*+1, *y*+1/2, −*z*+3/2.
